# Advanced Strategies in Immune Modulation of Cancer Using Lipid-Based Nanoparticles

**DOI:** 10.3389/fimmu.2017.00069

**Published:** 2017-02-06

**Authors:** Shoshy Mizrahy, Inbal Hazan-Halevy, Dalit Landesman-Milo, Brandon D. Ng, Dan Peer

**Affiliations:** ^1^Laboratory of Precision NanoMedicine, Department of Cell Research and Immunology, George S. Wise Faculty of Life Sciences, Tel Aviv University, Tel Aviv, Israel; ^2^Department of Materials Sciences and Engineering, Iby and Aladar Fleischman Faculty of Engineering, Tel Aviv University, Tel Aviv, Israel; ^3^Center for Nanoscience and Nanotechnology, Tel Aviv University, Tel Aviv, Israel; ^4^Cancer Biology Research Center, Tel Aviv University, Tel Aviv, Israel

**Keywords:** lipid nanoparticles, cancer immunotherapy, siRNA therapeutic, tumor microenvironment, cancer vaccines

## Abstract

Immunotherapy has a great potential in advancing cancer treatment, especially in light of recent discoveries and therapeutic interventions that lead to complete response in specific subgroups of melanoma patients. By using the body’s own immune system, it is possible not only to specifically target and eliminate cancer cells while leaving healthy cells unharmed but also to elicit long-term protective response. Despite the promise, current immunotherapy is limited and fails in addressing all tumor types. This is probably due to the fact that a single treatment strategy is not sufficient in overcoming the complex antitumor immunity. The use of nanoparticle-based system for immunotherapy is a promising strategy that can simultaneously target multiple pathways with the same kinetics to enhance antitumor response. Here, we will highlight the recent advances in the field of cancer immunotherapy that utilize lipid-based nanoparticles as delivery vehicles and address the ongoing challenges and potential opportunities.

## Introduction

### Tumor Immunology

Hundred years after Paul Ehrlich coined the term “magic bullet,” it is well established that the immune system can be utilized in the epic battle against cancer. Immune cells possess a unique ability to distinguish between cancer and normal cells with reliance on specific expression of cell-surface tumor-associated antigens (TAA) or self-antigens. Due to this ability, immune cells can act as “killing bullets” that are able to specifically eliminate tumors cells.

The development of a tumor is a dynamic process whereby the immune system not only protects against cancer development but also shapes the character of emerging tumors, a process named “Cancer Immunoediting.” This complex process can be divided to three phases according to the nature of the interaction of tumor cells with the immune system: elimination, equilibrium, and escape (1).

In the elimination phase, both the innate and adaptive immune systems detect and destroy early tumors (2). Tumor cells express TAA-derived peptides in context of MHC molecules that are recognized by TAA-specific-cytotoxic CD8^+^T-lymphocytes (CTL). Activated CTL and natural killer (NK) cells directly induce tumor cell apoptosis and, in addition, release IFN-γ that mediates inhibition of tumor-cell proliferation and angiogenesis. Antigen-presenting cells (APCs), such as tumor-associated macrophages (TAMs) and dendritic cells (DC), take up TAAs from tumor cells debris and present their peptides in the context of MHC to TAA-specific CD8^+^ and CD4^+^T-lymphocytes. Activated CD4^+^T-lymphocytes produce inflammatory cytokines such as, IFN-γ and TNF-α, that can both suppress tumor survival and upregulate the expression of MHC molecules on the surface of tumor cells. This upregulation facilitates the recognition of cancer cells by CTL, which mediates their killing mechanism (1, 3).

The equilibrium phase is a balance phase upon which tumor progression is still controlled by the immune system; however, sporadic tumor cells manage to survive immune destruction (1, 4).

In the escape phase, tumors manage to evade immune surveillance, begin to grow progressively and establish an immunosuppressive tumor microenvironment (TME) (1, 5, 6). In this phase, tumor cells acquire the ability to escape from host immune response through different strategies; first, tumor cells downregulate MHC expression, thus preventing recognition and attack by CTL (7). Second, tumor cells upregulate their expression of immune checkpoints proteins, such as PD-L1 and CD80/CD86, which ligate the negative co-stimulators PD1 and CTLA4 on the T-lymphocyte’s membrane. This results in attenuation of T-lymphocytes activation (8, 9). Furthermore, tumor cells induce the infiltration of other cell types (fibroblasts, endothelial, and immune cells) and instruct them to establish TME that promotes tumor progression (10). For example, infiltration of regulatory T-cells (Tregs) into the TME inhibits CTL (11). Finally, tumor cells encourage cells in TME to release anti-inflammatory cytokines, such as IL-10 and TGF-β. These cytokines suppress the ability of resident immune cells to act against tumor cells, prevent the recruitment of CTL and NK cells, and, therefore, promote tumor growth (12).

In the past few years, the rapidly advancing field of cancer immunotherapy has produced several new opportunities for treating cancer: either by stimulating the activities of specific components of the immune system or by counteracting immunosuppressive signals produced by tumor cells. Efficient antitumor immunotherapy can be achieved by combining delivery of TAAs to APC along with removing tumor-derived negative regulators of immune cell activation (13, 14). Therefore, delivery of immunomodulatory molecules to the appropriate cells is crucial for the successful development of cell-based antitumor immunotherapy.

### Nanotechnology-Based Therapies

Nanotechnology affords a unique opportunity to deliver therapeutic molecules to specific cells and simultaneously attack several biological avenues promoting tumor eradication (15). A variety of delivery platform vehicles are under development to target immune cells, such as antibodies (16), polymers (17), aptamers (18), and lipid nanoparticles (LNPs) (19, 20). In comparison to other NPs, LNPs have attractive biological properties, which include general biocompatibility, biodegradability, and the ability to entrap both hydrophilic and hydrophobic drugs (15, 21). Multiple properties of LNPs can be altered *via* different lipid composition and ratios or by surface chemistry, including their size, charge, and surface functionality (22). LNPs serve as carriers for a variety of therapeutic molecules: from nucleic acid to proteins, small molecules and chemotherapy drugs, and combinations of the aforementioned agents (22–24). LNPs shelter their cargo from clearance in the surrounding biological milieu, increasing the half-life in circulation, minimizing systemic toxicity, and hence enabling a wide therapeutic window (15, 22, 25). In addition, LNPs can promote delivery of their cargo directly to specific immune cell, such as APC or T-lymphocytes (20, 26, 27). Here, we review the latest developments in nanoparticle-based cancer immunotherapy, centering on LNPs.

## The Effects of the Physicochemical Properties of LNPs on Immune Modulation

The physicochemical characteristics of LNPs are crucial for their fate and performance following administration. Several parameters were examined including nanoparticle size, shape, surface charge, lipid composition, and aggregation. Size is one of the major causes for immune stimulation by LNPs. This can be related to the fact that these nanoparticles fall within the size range of pathogens, such as viruses and small bacteria (28). Size has a significant impact on blood circulation half-life as an optimal vesicle should be large enough to avoid renal clearance but small enough to avoid clearance *via* the mononuclear phagocytic system (15). The size of NPs should be also adjusted in accordance to the route of administration and delivery purposes. For example, it was shown that, following intravenous administration, 25 nm size NPs are significantly more efficient than 100 nm-sized NPs, as these smaller nanoparticles are better delivered to the lymph nodes (29). In addition, size of below 100 nm enables NPs to utilize the enhanced permeability and retention effect characteristic of solid tumors. Below 100 nm, NPs can reach solid tumors by extravasation *via* the leaky tumor vasculature and accumulate due to poor lymphatic drainage (15).

The shape and curvature of NPs can also affect immune activation as it was shown that oblate-shaped NPs have a lower macrophage uptake in comparison to spherical nanoparticles and, therefore, longer circulation time and altered biodistribution (30).

Surface features of LNPs have also been thoroughly investigated. Surface charge is another major contributor of immune activation, as positively charged LNPs can better interact with the negatively charged mucosal surface. This results in better uptake of positively charged LNPs by cells in comparison to their negatively charged or neutral counterparts (31). However, positively charged LNPs have toxic effects including induction of pro-inflammatory response (32). Surface charge (both negative and positive) and high membrane cholesterol content are also related to complement activation by liposomes (28). Contrarily, the presence of phosphatidylserine (PS) has an anti-inflammatory effect probably due to specific recognition by PS receptors (33, 34).

Surface functionalization has been widely used to improve LNPs performance. One of the main causes for the immune recognition of LNPs is the absence of self-discriminating molecules (such as complement control proteins) on the bilayer membrane, which protect “self” cells from attack by the complement system (28). Among the most common of these are hydrophilic moieties, such as polyethylene glycol (PEG), which enables prolonged blood circulation time and improved biodistribution by protecting NPs from opsonization (15). Despite the multiple advantages, functionalizion with PEG can induce complement activation (5, 35, 36). In addition, subsequent injections of PEGylated liposomes can result in their accelerated blood clearance, probably due to transient IgM production (37). Another option for hydrophilic surface coating is polysaccharides. These naturally occurring molecules are a great alternative as they are characterized by low toxicity, low immunogenicity, biocompatibility, stability, low cost, cryoprotection, and availability of reactive sites for chemical modification (38). A specific example is hyaluronan (HA), which is also characterized by bioadhesive properties as part of the extracellular matrix. In addition, HA is the major ligand for CD44 and CD168 receptors and, therefore, suitable for specific targeting to cells, such as those in tumors that overexpress these receptors (39–42).

Additional surface functionalization includes ligands that enable specific cell targeting, such as antibodies, peptides, and aptamers (15). However, despite the advantage, LNPs decorated with proteins or peptides can elicit an unwanted immune response, cross link receptors, and generate an outside-in signaling cascade (43–45).

There are additional factors that induce immune activation of LNPs; among these are inhomogeneity of size or shape, multilamellar structure, endotoxin contamination, aggregation, and the presence of non-encapsulated drugs that can bind and aggregate lipids. Ultimately, it seems that the longest circulation time is obtained using LNPs that better mimic natural membranes. Therefore, there are increasing efforts to create LNPs using natural materials and even natural membrane-derived LNPs. Recently, Rodriguez et al. have shown that surface modification of LNPs with a “self” peptide segment of CD47 resulted in reduced blood clearance by macrophages and, therefore, longer circulation time (46, 47).

The vast progress in the field of drug delivery enables tailoring specific NPs for a particular location (TME, lymph nodes) and cell type while avoiding unwanted non-specific immune responses and toxicities as detailed below.

## LNP-Based Immunotherapies

### Immunomodulation of APCs

**Figure 1:**
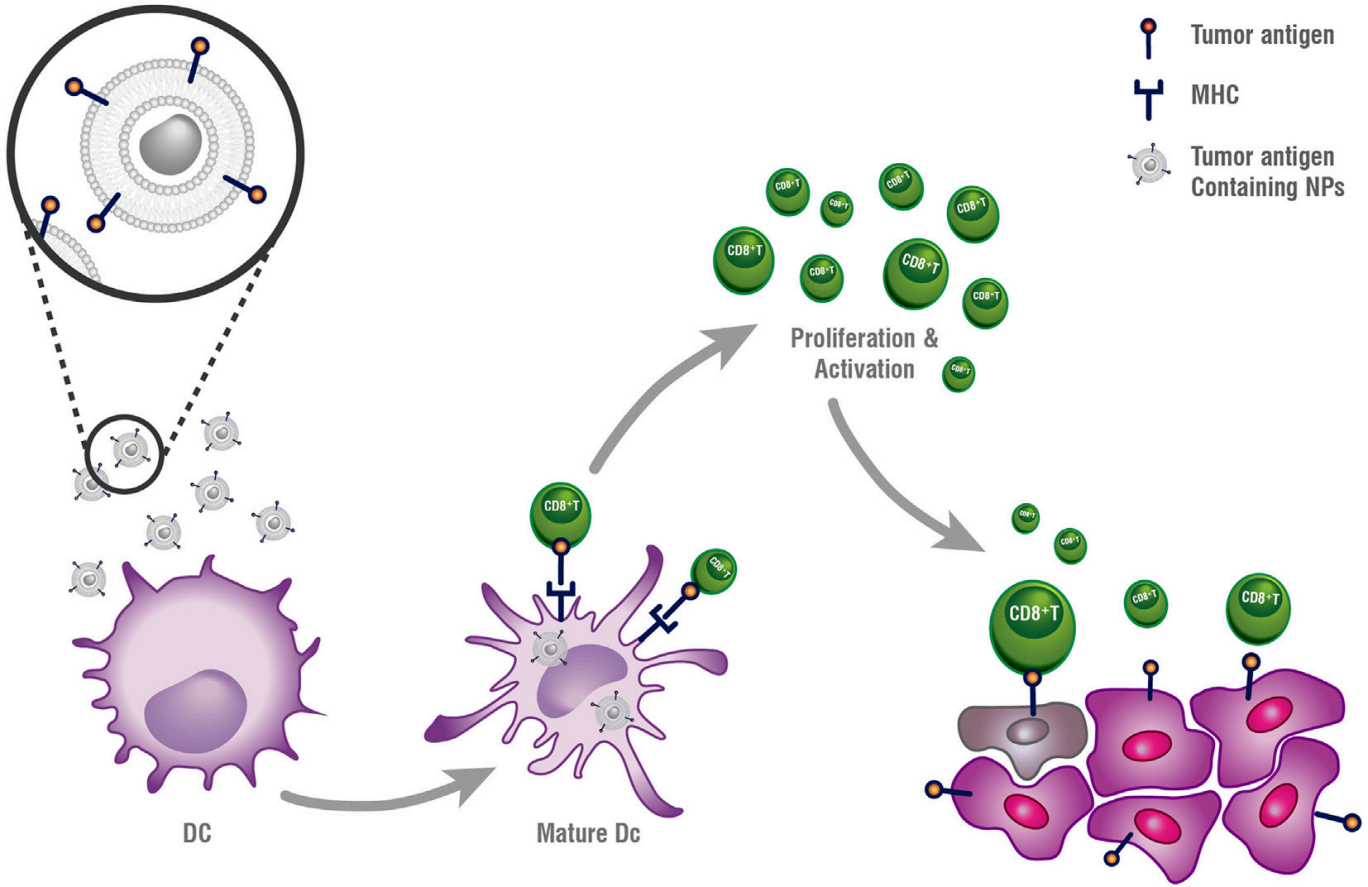
**Antitumor vaccine**. Lipid nanoparticles (LNPs) can be loaded with tumor antigens and adjuvants for the purpose of targeting antigen-presenting cells, such as dendritic cells (DCs). Following uptake, the LNPs degrade and release the antigen and adjuvant, which stimulates DC maturation and antigen presenting on cell-surface MHC molecules. This context enables recognition and binding of CD8^+^ T-lymphocytes and results in their activation, proliferation, and antitumor response.

Therapeutic anticancer vaccination is a strategy that attempts to improve host immunity to cancer by upregulating host immune response against TAAs. Tumor vaccines deliver the antigen and/or adjuvant to APCs for the activation of both humoral and cellular immunity as presented in Figure 1.

NP delivery can significantly boost immunogenicity of tumor antigens by co-delivery of antigens and adjuvants to the same location (48). One such platform is interbilayer-crosslinked multilamellar vesicles (ICMVs), which entrap high amounts of protein antigens in the vesicle core and lipid-based adjuvant in the vesicle walls (49). The authors found that ICMVs elicited robust antibody titers greater than simple liposomes or multi lamellar vesicles of identical lipid compositions. These synthetic vesicles triggered steadily increasing antibody production and CTL responses following repeated administrations.

Several NP-based vaccine formulations have been developed to deliver antigens specifically to APCs, especially to DCs and TAMs. Recently, Kranz et al. (50) used RNA-lipoplexes (RNA-LPX), which are LNPs encapsulated with an RNA-encoded antigen, for systemic targeting of DCs. Targeting the DCs by RNA-LPX is based on optimally adjusting the net charge of the LNPs without functionalization of particles. RNA-LPX encoding antigens induce strong effector and memory T-cell responses and mediate potent IFNα-dependent rejection of progressive tumors. Leuschner et al. developed LNPs platform encapsulated with siRNA for modulation of monocytes and macrophages (51). The authors used this platform to encapsulate siRNA against CCR2, an important monocyte homing factor, and tested it in a mouse lymphoma model. Systemic administration of these nanoparticles resulted in a lower numbers of TAMs and reduction of tumor size.

Others have shown targeting of DCs using NPs decorated by specific anti DC antibodies. For example, Macho-Fernandez et al. (52) used NPs generated from poly lactic-*co*-glycolic acid (PLGA), coated with lipid-PEG and decorated by Abs recognizing DEC205, a cell-surface receptor that is expressed on spleen and lymph node CD8^+^ DCs. Using these NPs, they have demonstrated that co-delivery of an agonist (a-GalCer) and a protein antigen (ovalbumin) to CD8^+^ DCs triggers optimal humoral and CTL responses. In addition, this platform promotes potent antitumor responses in a B16F10 murine melanoma tumor model.

### Immunomodulation of Tumor Cells and TME

Over time, cancer cells can develop phenotypes that are less immunogenic in order to escape immune surveillance. One strategy used by cancer cells is an elevated expression of self-markers to avoid immune recognition by professional phagocytes. An example of using this strategy is the over expression of CD47 on the cancer cell surface, which labels the cells with the “self” marker and correlates with poor clinical prognosis (53–55). Yang et al. developed a systemic delivery strategy based on CD47 siRNA encapsulated in HA-coated LNPs, which led to an efficient knockdown of CD47 in cancer cells. Decreased expression of CD47 eventually led to growth inhibition of melanoma tumors and suppressed lung metastasis in a B16F10 murine melanoma tumor model (56).

Additional strategy used by cancer cells to avoid immune recognition is secretion of anti-inflammatory cytokines. Anti-inflammatory cytokines prevent the recruitment of immune cells to the TME and suppress the ability of resident immune cells to act against tumor cells. In order to block these complex interactions, immune modulation based on targeting both the tumor cells and DCs was developed by Xu et al. (57). They delivered a combination of NPs: liposome–protamine–HA NPs encapsulated with siRNA against the immune-suppressive cytokine TGF-β, and mannose-modified lipid–calcium–phosphate NPs encapsulated with tumor antigen and adjuvant. TGF-β downregulation boosted the vaccine efficacy and inhibited tumor growth, as a result of increased levels of tumor infiltrating CD8^+^ T-lymphocytes and decreased level of Tregs.

**Figure 2:**
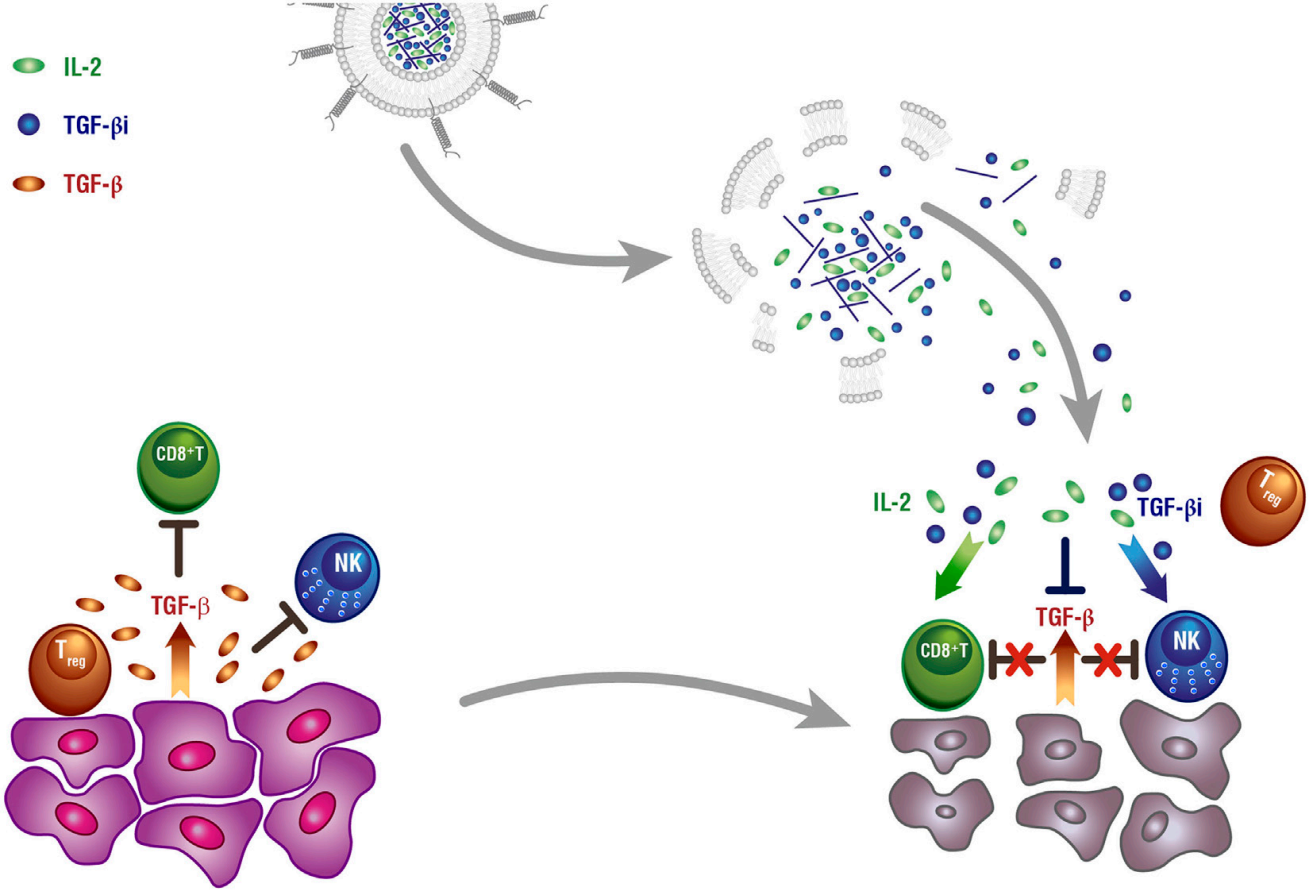
**Lipid nanoparticle (LNP)-based cytokine remodeling of tumor microenvironment (TME)**. The TME manages to escape immune surveillance *via* production of immunosuppressive cytokines, such as TGF-β and IL-10. TGF-β inhibits both innate and adaptive immune responses by suppressing the activity of CD8^+^T-lymphocytes (CTL) and natural killer (NK) cells as well as triggering the expansion of Tregs. LNPs entrapping both TGF-β inhibitor and IL-2 manage to reverse this effect by directly activating both NK and CTL, while also depleting Tregs; thus restoring both adaptive and innate antitumor responses [modified from Ref. (60)]. Reproduced by permission from Macmillan Publishers Ltd: Nature Materials, 11, 831–832 (2012), copyright 2012.

A liposomal polymeric gel system developed by Park et al., which combines the delivery of TGF-β inhibitor and the pro-inflammatory cytokine IL-2, is another example for a complex approach of immunomodulation (Figure 2) (58). IL-2 was shown to enhance NK and CTL activity against several cancer types; however, this ability is hampered due to the secretion of anti-inflammatory agents, such as TGF-β by tumor cells (59). The authors showed a significant reduction of tumor growth *in vivo* and an increased immune response.

Another example of combining several immunotherapy strategies was recently presented by Moynihan et al. (61). The authors used the amphiphilic vaccine platform containing a tumor antigen and adjuvant conjugated to albumin binding lipid. This enables utilizing the “albumin hitchhiking” strategy upon which binding with albumin directly targets molecules to lymphatics and draining LNs, thereby allowing them to accumulate in APCs. This platform was combined with systemic administration of tumor antigen-specific antibodies, IL-2, and anti- PD-1 antibodies. The authors showed recruitment of both innate and adaptive immune cells that led to elimination of large established tumor burdens in syngeneic and genetically engineered murine tumor models. The treatment also elicited long protective T cell memory response.

## Sequential Treatment of Chemotherapy Followed by Immunotherapy

A combined chemo-immunotherapeutic approach may be beneficial for the efficient elimination of cancer. Chemotherapy eliminates tumor cells, causing the cancer to shrink. As a result, tumor-derived antigens, such as peptides or proteins isolated from the tumor cells, can be efficiently internalized by APCs, thereby increasing the antitumor immunity of CTL. Lu et al. (62) developed cisplatin LNPs and CpG-encapsulated liposomes for treatment of melanoma. Such combination therapy established strong synergistic effects, both on the apoptotic level and subsequent abrogation of tumor growth. Heo et al. (63) sequentially subjected tumor-bearing mice to chemotherapy consisting of pacitaxel dissolved in HA in addition to immunotherapy using CpG ODNs and IL-10 siRNA incorporated into PLGA NPs. The sequential treatment with chemotherapeutic drugs followed by a combined immunostimulation strategy resulted in a synergistic effect against solid tumors.

A recent study by He et al. designed coordination polymer LNPs, which combines two therapeutic modalities, chemotherapy and photodynamic therapy, to elicit antitumor immunity (64). These LNPs kill cancer cells by inducing apoptosis and necrosis, stimulating host immune system, and causing an acute inflammation and leukocyte infiltration to the tumors; all of which increase the presentation of tumor-derived antigens to T cells. Combining polymer NPs treatment with PD-L1 checkpoint blockade therapy led to the regression of the primary tumors treated locally with irradiation, and also resulted in the regression of the distant tumors in bilateral syngeneic mouse tumor models of CT26 and MC38.

## Conclusion and Future Outlook

Cancer immunotherapy has been getting a lot of attention in the past few years and for many good reasons. There are multiple benefits for immunotherapy in comparison to conventional medicine, as the former treatment modality enables utilization of the immune system to eliminate tumor cells, while leaving healthy cells untouched. As our understanding of the complex interplay between cancer cells and the immune system deepens, the potential for achieving efficient therapeutics grows. Advances in research in the past few years have provided a solid basis for the development of several therapeutic strategies on the basis of targeting specific pathways and checkpoints. Further understanding of the immunosuppressive TME and antitumor immunity is the key for successful treatments and avoiding unfavorable outcome such as induction of autoimmunity.

NP-based immunotherapy provides multiple advantages upon administration of immune modulators since it enables targeted delivery both locally and temporally, therefore enhancing the effectiveness and reducing toxicity. It also enables transport of several compounds simultaneously and delivery of RNAi-based therapeutics, significantly increasing the therapeutic index.

A successful immunotherapy requires both innate and adoptive responses. Tumor cells utilize several mechanisms to escape immune recognition and induce immune-suppressive TME; thus, tackling only one pillar would not be sufficient. This may be responsible for the limited clinical achievements of current immunotherapy. Indeed, recent treatments combining several immune effectors were reported to be synergistic. The identification of the optimal combination of NPs and immune modulators for the appropriate TME is also crucial, and further development may be aided by the use of computerized biology. Improvement of currently used murine tumor models to appropriately reflect the complex TME is also required. Many reports are treating early-stage tumors in which the immune-suppressive TME is not completely developed. In addition, direct manipulation of tumor-residing lymphocytes has not yet been achieved and the appropriate delivery system is still an unmet need. This is especially significant in light of the recently approved anti T-lymphocyte treatments (anti-CTLA-4 and anti-PD-1 antibodies) that emphasize the potential of T-lymphocyte modulation for immunotherapy. Currently, lymphocyte manipulation can be achieved indirectly *via* specific modulators; and direct targeting of specific subsets of APCs has already been demonstrated. Some reports even show this simply by controlling lipid composition or charge, avoiding the use of targeting moieties altogether. The effectiveness of such untargeted systems in humans still has yet to be determined. To date, impressive therapeutic effects have been achieved upon using adoptive cell transfer therapy; however, this approach is not feasible for large-scale treatment. Therefore, the optimal NP-based system should include the appropriate immune mediator combination that would elicit highly effective and wholly endogenous response.

## Author Contributions

SM, IH-H, DL-M, and BN wrote the manuscript. DP wrote and edited the manuscript.

## Conflict of Interest Statement

DP declares a financial interest in Quiet Therapeutics. The other authors declare no conflict of interest.

## References

[1] Mittal D Gubin MM Schreiber RD Smyth MJ New insights into cancer immunoediting and its three component phases – elimination, equilibrium and escape. Curr Opin Immunol (2014) 27:16–25. 10.1016/j.coi.2014.01.004 24531241 PMC4388310

[2] Kapadia CH Perry JL Tian S Luft JC DeSimone JM. Nanoparticulate immunotherapy for cancer. J Control Release (2015) 219:167–80. 10.1016/j.jconrel.2015.09.062 26432555

[3] Gajewski TF Schreiber H Fu YX. Innate and adaptive immune cells in the tumor microenvironment. Nat Immunol (2013) 14:1014–22. 10.1038/ni.2703 24048123 PMC4118725

[4] Dunn GP Old LJ Schreiber RD. The immunobiology of cancer immunosurveillance and immunoediting. Immunity (2004) 21:137–48. 10.1016/j.immuni.2004.07.017 15308095

[5] Hanahan D Weinberg RA Hallmarks of cancer: the next generation. Cell (2011) 144:646–74. 10.1016/j.cell.2011.02.013 21376230

[6] Vinay DS Ryan EP Pawelec G Talib WH Stagg J Elkord E Immune evasion in cancer: mechanistic basis and therapeutic strategies. Semin Cancer Biol (2015) 35:S185–98. 10.1016/j.semcancer.2015.03.004 25818339

[7] Wu JD Higgins LM Steinle A Cosman D Haugk K Plymate SR. Prevalent expression of the immunostimulatory MHC class I chain-related molecule is counteracted by shedding in prostate cancer. J Clin Invest (2004) 114:560–8. 10.1172/JCI200422206 15314693 PMC503776

[8] Gribben JG Freeman GJ Boussiotis VA Rennert P Jellis CL Greenfield E CTLA4 mediates antigen-specific apoptosis of human T cells. Proc Natl Acad Sci U S A (1995) 92:811–5. 10.1073/pnas.92.3.811 7846057 PMC42710

[9] Iwai Y Ishida M Tanaka Y Okazaki T Honjo T Minato N. Involvement of PD-L1 on tumor cells in the escape from host immune system and tumor immunotherapy by PD-L1 blockade. Proc Natl Acad Sci U S A (2002) 99:12293–7. 10.1073/pnas.192461099 12218188 PMC129438

[10] Goubran HA Kotb RR Stakiw J Emara ME Burnouf T. Regulation of tumor growth and metastasis: the role of tumor microenvironment. Cancer Growth Metastasis (2014) 7:9–18. 10.4137/CGM.S11285 24926201 PMC4051818

[11] Khazaie K von Boehmer H. The impact of CD4+CD25+ Treg on tumor specific CD8+ T cell cytotoxicity and cancer. Semin Cancer Biol (2006) 16:124–36. 10.1016/j.semcancer.2005.11.006 16443370

[12] Tang X Mo C Wang Y Wei D Xiao H. Anti-tumour strategies aiming to target tumour-associated macrophages. Immunology (2013) 138:93–104. 10.1111/imm.12023 23113570 PMC3575762

[13] Vanneman M Dranoff G. Combining immunotherapy and targeted therapies in cancer treatment. Nat Rev Cancer (2012) 12:237–51. 10.1038/nrc3237 22437869 PMC3967236

[14] Pardoll DM. The blockade of immune checkpoints in cancer immunotherapy. Nat Rev Cancer (2012) 12:252–64. 10.1038/nrc3239 22437870 PMC4856023

[15] Peer D Karp JM Hong S Farokhzad OC Margalit R Langer R. Nanocarriers as an emerging platform for cancer therapy. Nat Nanotechnol (2007) 2:751–60. 10.1038/nnano.2007.387 18654426

[16] Song E Zhu P Lee SK Chowdhury D Kussman S Dykxhoorn DM Antibody mediated in vivo delivery of small interfering RNAs via cell-surface receptors. Nat Biotechnol (2005) 23:709–17. 10.1038/nbt1101 15908939

[17] Chou ST Mixson AJ siRNA nanoparticles: the future of RNAi therapeutics for oncology? Nanomedicine (Lond) (2014) 9:2251–4. 10.2217/nnm.14.157 25413851 PMC4788000

[18] Wheeler LA Trifonova R Vrbanac V Basar E McKernan S Xu Z Inhibition of HIV transmission in human cervicovaginal explants and humanized mice using CD4 aptamer-siRNA chimeras. J Clin Invest (2011) 121:2401–12. 10.1172/JCI45876 21576818 PMC3104760

[19] Peer D. A daunting task: manipulating leukocyte function with RNAi. Immunol Rev (2013) 253:185–97. 10.1111/imr.12044 23550647

[20] Ramishetti S Kedmi R Goldsmith M Leonard F Sprague AG Godin B Systemic gene silencing in primary T lymphocytes using targeted lipid nanoparticles. ACS Nano (2015) 9:6706–16. 10.1021/acsnano.5b02796 26042619

[21] Bunjes H Lipid nanoparticles for the delivery of poorly water-soluble drugs. J Pharm Pharmacol (2010) 62:1637–45. 10.1111/j.2042-7158.2010.01024.x 21039547

[22] Torchilin VP. Recent advances with liposomes as pharmaceutical carriers. Nat Rev Drug Discov (2005) 4:145–60. 10.1038/nrd1632 15688077

[23] Kanasty R Dorkin JR Vegas A Anderson D. Delivery materials for siRNA therapeutics. Nat Mater (2013) 12:967–77. 10.1038/nmat3765 24150415

[24] Yin H Kanasty RL Eltoukhy AA Vegas AJ Dorkin JR Anderson DG. Non-viral vectors for gene-based therapy. Nat Rev Genet (2014) 15:541–55. 10.1038/nrg3763 25022906

[25] Torchilin VP. Multifunctional, stimuli-sensitive nanoparticulate systems for drug delivery. Nat Rev Drug Discov (2014) 13:813–27. 10.1038/nrd4333 25287120 PMC4489143

[26] Shao K Singha S Clemente-Casares X Tsai S Yang Y Santamaria P. Nanoparticle-based immunotherapy for cancer. ACS Nano (2015) 9:16–30. 10.1021/nn5062029 25469470

[27] Smith DM Simon JK Baker JR Jr Applications of nanotechnology for immunology. Nat Rev Immunol (2013) 13:592–605. 10.1038/nri3517 23883969 PMC7097370

[28] Szebeni J Barenholz YC Chapter 11: Complement activation, immunogenicity, and immune suppression as potential side effects of liposomes. In: Peer D, editor. Handbook of Harnessing Biomaterials in Nanomedicine – Preparation, Toxicity and Applications. Singapore: Pan Stanford (2012). p. 309–35.

[29] Reddy ST van der Vlies AJ Simeoni E Angeli V Randolph GJ O’Neil CP Exploiting lymphatic transport and complement activation in nanoparticle vaccines. Nat Biotechnol (2007) 25:1159–64. 10.1038/nbt1332 17873867

[30] Toy R Peiris PM Ghaghada KB Karathanasis E. Shaping cancer nanomedicine: the effect of particle shape on the in vivo journey of nanoparticles. Nanomedicine (Lond) (2014) 9:121–34. 10.2217/nnm.13.191 24354814 PMC4057606

[31] Verma A Stellacci F Effect of surface properties on nanoparticle-cell interactions. Small (2010) 6:12–21. 10.1002/smll.200901158 19844908

[32] Kedmi R Ben-Arie N Peer D. The systemic toxicity of positively charged lipid nanoparticles and the role of toll-like receptor 4 in immune activation. Biomaterials (2010) 31:6867–75. 10.1016/j.biomaterials.2010.05.027 20541799

[33] Fadok VA Bratton DL Rose DM Pearson A Ezekewitz RA Henson PM. A receptor for phosphatidylserine-specific clearance of apoptotic cells. Nature (2000) 405:85–90. 10.1038/35011084 10811223

[34] Ramos GC Fernandes D Charao CT Souza DG Teixeira MM Assreuy J. Apoptotic mimicry: phosphatidylserine liposomes reduce inflammation through activation of peroxisome proliferator-activated receptors (PPARs) in vivo. Br J Pharmacol (2007) 151:844–50. 10.1038/sj.bjp.0707302 17533418 PMC2014119

[35] Moghimi SM Hamad I Andresen TL Jorgensen K Szebeni J. Methylation of the phosphate oxygen moiety of phospholipid-methoxy(polyethylene glycol) conjugate prevents PEGylated liposome-mediated complement activation and anaphylatoxin production. FASEB J (2006) 20:2591–3. 10.1096/fj.06-6186fje 17065229

[36] Szebeni J Baranyi L Savay S Milosevits J Bunger R Laverman P Role of complement activation in hypersensitivity reactions to doxil and hynic PEG liposomes: experimental and clinical studies. J Liposome Res (2002) 12:165–72. 10.1081/LPR-120004790 12604051

[37] Dams ET Laverman P Oyen WJ Storm G Scherphof GL van Der Meer JW Accelerated blood clearance and altered biodistribution of repeated injections of sterically stabilized liposomes. J Pharmacol Exp Ther (2000) 292:1071–9. 10688625

[38] Mizrahy S Peer D. Polysaccharides as building blocks for nanotherapeutics. Chem Soc Rev (2012) 41:2623–40. 10.1039/c1cs15239d 22085917

[39] Cohen K Emmanuel R Kisin-Finfer E Shabat D Peer D. Modulation of drug resistance in ovarian adenocarcinoma using chemotherapy entrapped in hyaluronan-grafted nanoparticle clusters. ACS Nano (2014) 8:2183–95. 10.1021/nn500205b 24494862

[40] Cohen ZR Ramishetti S Peshes-Yaloz N Goldsmith M Wohl A Zibly Z Localized RNAi therapeutics of chemoresistant grade IV glioma using hyaluronan-grafted lipid-based nanoparticles. ACS Nano (2015) 9:1581–91. 10.1021/nn506248s 25558928

[41] Mizrahy S Goldsmith M Leviatan-Ben-Arye S Kisin-Finfer E Redy O Srinivasan S Tumor targeting profiling of hyaluronan-coated lipid based-nanoparticles. Nanoscale (2014) 6:3742–52. 10.1039/c3nr06102g 24569711

[42] Yerushalmi N Arad A Margalit R. Molecular and cellular studies of hyaluronic acid-modified liposomes as bioadhesive carriers for topical drug delivery in wound healing. Arch Biochem Biophys (1994) 313:267–73. 10.1006/abbi.1994.1387 8080272

[43] Reissmann S. Cell penetration: scope and limitations by the application of cell-penetrating peptides. J Pept Sci (2014) 20:760–84. 10.1002/psc.2672 25112216

[44] Peer D Shimaoka M. Systemic siRNA delivery to leukocyte-implicated diseases. Cell Cycle (2009) 8:853–9. 10.4161/cc.8.6.7936 19221492

[45] Imai Y Park EJ Peer D Peixoto A Cheng G von Andrian UH Genetic perturbation of the putative cytoplasmic membrane-proximal salt bridge aberrantly activates alpha(4) integrins. Blood (2008) 112:5007–15. 10.1182/blood-2008-03-144543 18809756 PMC2597606

[46] Oldenborg PA Zheleznyak A Fang YF Lagenaur CF Gresham HD Lindberg FP. Role of CD47 as a marker of self on red blood cells. Science (2000) 288:2051–4. 10.1126/science.288.5473.2051 10856220

[47] Rodriguez PL Harada T Christian DA Pantano DA Tsai RK Discher DE Minimal “self” peptides that inhibit phagocytic clearance and enhance delivery of nanoparticles. Science (2013) 339:971–5. 10.1126/science.1229568 23430657 PMC3966479

[48] Goldberg MS. Immunoengineering: how nanotechnology can enhance cancer immunotherapy. Cell (2015) 161:201–4. 10.1016/j.cell.2015.03.037 25860604

[49] Moon JJ Suh H Bershteyn A Stephan MT Liu H Huang B Interbilayer-crosslinked multilamellar vesicles as synthetic vaccines for potent humoral and cellular immune responses. Nat Mater (2011) 10:243–51. 10.1038/nmat2960 21336265 PMC3077947

[50] Kranz LM Diken M Haas H Kreiter S Loquai C Reuter KC Systemic RNA delivery to dendritic cells exploits antiviral defence for cancer immunotherapy. Nature (2016) 534:396–401. 10.1038/nature18300 27281205

[51] Leuschner F Dutta P Gorbatov R Novobrantseva TI Donahoe JS Courties G Therapeutic siRNA silencing in inflammatory monocytes in mice. Nat Biotechnol (2011) 29:1005–10. 10.1038/nbt.1989 21983520 PMC3212614

[52] Macho-Fernandez E Cruz LJ Ghinnagow R Fontaine J Bialecki E Frisch B Targeted delivery of alpha-galactosylceramide to CD8alpha+ dendritic cells optimizes type I NKT cell-based antitumor responses. J Immunol (2014) 193:961–9. 10.4049/jimmunol.1303029 24913977

[53] Chan KS Espinosa I Chao M Wong D Ailles L Diehn M Identification, molecular characterization, clinical prognosis, and therapeutic targeting of human bladder tumor-initiating cells. Proc Natl Acad Sci U S A (2009) 106:14016–21. 10.1073/pnas.0906549106 19666525 PMC2720852

[54] Jaiswal S Jamieson CH Pang WW Park CY Chao MP Majeti R CD47 is upregulated on circulating hematopoietic stem cells and leukemia cells to avoid phagocytosis. Cell (2009) 138:271–85. 10.1016/j.cell.2009.05.046 19632178 PMC2775564

[55] Majeti R Chao MP Alizadeh AA Pang WW Jaiswal S Gibbs KD Jr CD47 is an adverse prognostic factor and therapeutic antibody target on human acute myeloid leukemia stem cells. Cell (2009) 138:286–99. 10.1016/j.cell.2009.05.045 19632179 PMC2726837

[56] Wang Y Xu Z Guo S Zhang L Sharma A Robertson GP Intravenous delivery of siRNA targeting CD47 effectively inhibits melanoma tumor growth and lung metastasis. Mol Ther (2013) 21:1919–29. 10.1038/mt.2013.135 23774794 PMC3808133

[57] Xu Z Wang Y Zhang L Huang L Nanoparticle-delivered transforming growth factor-beta siRNA enhances vaccination against advanced melanoma by modifying tumor microenvironment. ACS Nano (2014) 8:3636–45. 10.1021/nn500216y 24580381 PMC4004320

[58] Park J Wrzesinski SH Stern E Look M Criscione J Ragheb R Combination delivery of TGF-beta inhibitor and IL-2 by nanoscale liposomal polymeric gels enhances tumour immunotherapy. Nat Mater (2012) 11:895–905. 10.1038/nmat3355 22797827 PMC3601683

[59] Wrzesinski SH Wan YY Flavell RA. Transforming growth factor-beta and the immune response: implications for anticancer therapy. Clin Cancer Res (2007) 13:5262–70. 10.1158/1078-0432.CCR-07-1157 17875754

[60] Brinker CJ Nanoparticle immunotherapy: combo combat. Nat Mater (2012) 11:831–2. 10.1038/nmat3434 23001226

[61] Moynihan KD Opel CF Szeto GL Tzeng A Zhu EF Engreitz JM Eradication of large established tumors in mice by combination immunotherapy that engages innate and adaptive immune responses. Nat Med (2016) 22:1402–10. 10.1038/nm.4200 27775706 PMC5209798

[62] Lu Y Wang Y Miao L Haynes M Xiang G Huang L. Exploiting in situ antigen generation and immune modulation to enhance chemotherapy response in advanced melanoma: a combination nanomedicine approach. Cancer Lett (2016) 379:32–8. 10.1016/j.canlet.2016.05.025 27235608

[63] Heo MB Kim SY Yun WS Lim YT. Sequential delivery of an anticancer drug and combined immunomodulatory nanoparticles for efficient chemoimmunotherapy. Int J Nanomedicine (2015) 10:5981–92. 10.2147/IJN.S90104 26451105 PMC4590313

[64] He C Duan X Guo N Chan C Poon C Weichselbaum RR Core-shell nanoscale coordination polymers combine chemotherapy and photodynamic therapy to potentiate checkpoint blockade cancer immunotherapy. Nat Commun (2016) 7:12499. 10.1038/ncomms12499 27530650 PMC4992065

